# The St. Neots Murder

**Published:** 1898-06-11

**Authors:** 


### The Hospital

A Journal of

The Medical Sciences and Hospital Administration.

Vol. XXIV.—No. 611.] Edited by Sir Henry Burdett, K.C.B., and Solomon C. Smith, M.D., M.R.C.P. [June 11, 1898.

### The St. Neots Murder.

The conviction of Walter Horsford on Monday last for the murder of his cousin, Annie Holmes, at St. Neots, must have come as a foregone conclusion to all who followed the course of the evidence given for the prosecution, and who noted the character of the defence. Upon the latter, indeed, it is hardly necessary to dwell, unless it be to join in Mr. Justice Hawkins's condemnation of the attempt made by counsel to obtain evidence by cross-examination of the deceased's daughter, a girl of fourteen, that her mother was a person of loose and profligate character. The existence of her young baby, and her admitted belief that she was again *enceinte*, afforded proof that she had overstepped propriety on more than one occasion; but this furnished no extenuation of the conduct of her murderer, nor was it in any way relevant to the issue before the jury, who were only called upon to consider the guilt or innocence of the prisoner. Regarded from this point of view, the case furnishes a remarkable example of the usefulness of the Sale of Poisons Act, but for which it is hardly conceivable that a conviction would have been obtained. It was only by police inquiries at the shops of chemists within a considerable radius that the possession of strychnia was brought home to the prisoner; and Thrapston, where the poison was bought, is several miles distant from his home at Spaldwick. The evidence of the chemist at Thrapston, who said it was "common for farmers to buy poison in this quantity," as well as that of Dr. Stevenson, who said that "it was by no means an uncommon thing for pregnant girls to die in this way, trying to procure abortion or to commit suicide," when taken together, are highly suggestive of a doubt whether the provisions of the Act might not with advantage be rendered even more stringent, and whether strychnia is not far too accessible to persons who may desire to use it for criminal purposes. A substance of which less than a single grain would be fatal to an average adult, and which cannot be used medicinally except with the greatest care, ought surely not to be procurable by any farmer who says that he wishes to poison rats, and who, in poisoning them, might by sheer carelessness easily bring about the sacrifice of human life as well. The destruction of rats, if it be indeed either called for or practised on a scale to render the purchase of ninety grains of strychnia a matter calling for no particular remark, should surely be undertaken only at stated periods, and by or under the direction of a pharmaceutical chemist, accustomed to deal with dangerous agents, and aware of the precautions by which their employment should be surrounded. In one respect, we cannot but hope that Dr. Stevenson's evidence, as reported, conveyed an impression somewhat in excess of the facts. We do not remember cases of strychnia poisoning among pregnant girls in sufficient number to justify what he is reported to have said; but, if he be correct, his statement would seem to call for a total prohibition of the sale of such a drug to any but those persons whose position would afford some legitimate reasons for desiring to use it.

Apart from the medico-legal aspects of the case, it is impossible to close the eyes to its repellant features in other directions, features which show that the life of rural districts does not always resemble that of an Arcadia. The relations between the prisoner and his not over scrupulous widowed cousin, the evident continuance of these relations notwithstanding his recent marriage, and the evident knowledge or suspicion of them on the part of his wife, all point to a state of society in which, if the schoolmaster has been abroad, there is still abundant work for the moralist and the preacher. Huntingdonshire does not occupy a very low position among English counties with regard to its proportion of illegitimate births; but the agricultural districts generally do not stand high in this particular, which may, perhaps, be taken as a fair test of public opinion with regard to matters of conduct. There is nothing to show that the poor woman who was murdered was regarded by her neighbours with disfavour, in spite of frailties which could not have been altogether concealed from them; and the presumption is that her case was not looked upon as an exceptional one. Her relatives seem to have been upon friendly terms with her, and there can be no doubt that her sad end has been an occasion for widely extended sympathy. The whole story is a melancholy comment upon the "who is she" which, in various forms, has presented itself as the central problem of crime to many who have been concerned in the work of detection. Sordid and vulgar though it be, the story none the less contains a moral, and teaches, like many other things, the facility of that descent to Avernus of which the poet told mankind in ages long bygone. It teaches, moreover, the latent possibilities of crime which underlie some of the most seemingly prosaic and humdrum of human existences. That a decent farmer, apparently of fair character and repute, should come to a disgraceful end upon the gallows, and leave the heritage of his shame to his family, is but the final consequence of an illicit connection with a woman who, as his near relative, should have been able to look to him for protection, even against herself.

